# Differentiation of Small Hepatocellular Carcinoma From Dysplastic Nodules in Cirrhotic Liver: Texture Analysis Based on MRI Improved Performance in Comparison Over Gadoxetic Acid-Enhanced MR and Diffusion-Weighted Imaging

**DOI:** 10.3389/fonc.2019.01382

**Published:** 2020-01-10

**Authors:** Xi Zhong, Hongsheng Tang, Bingui Lu, Jia You, Jinsong Piao, Peiyu Yang, Jiansheng Li

**Affiliations:** ^1^Department of Radiology, Affiliated Cancer Hospital & Institute of Guangzhou Medical University, Guangzhou, China; ^2^Department of Abdominal Surgery, Affiliated Cancer Hospital & Institute of Guangzhou Medical University, Guangzhou, China; ^3^Department of Pathology, Affiliated Cancer Hospital & Institute of Guangzhou Medical University, Guangzhou, China

**Keywords:** hepatocellular carcinoma, liver cirrhosis, diffusion magnetic resonance imaging, gadoxetic acid, texture analysis

## Abstract

**Background:** Accurate characterization of small (3 cm) hepatocellular carcinoma (sHCC) and dysplastic nodules (DNs) in cirrhotic liver is challenging. We aimed to investigate whether texture analysis (TA) based on T2-weighted images (T2WI) is superior to qualitative diagnosis using gadoxetic acid-enhanced MR imaging (Gd-EOB-MRI) and diffusion-weighted imaging (DWI) for distinguishing sHCC from DNs in cirrhosis.

**Materials and methods:** Sixty-eight patients with 73 liver nodules (46 HCCs, 27 DNs) pathologically confirmed by operation were included. For imaging diagnosis, three sets of images were reviewed by two experienced radiologists in consensus: a Gd-EOB-MRI set, a DWI set, and a combined set (combination of Gd-EOB-MRI and DWI). For TA, 279 texture features resulting from T2WI were extracted for each lesion. The performance of each approach was evaluated by a receiver operating characteristic analysis. The area under the receiver operating characteristic curve (*A*_z_), sensitivity, specificity, and accuracy were determined.

**Results:** The performance of TA (*A*_z_ = 0.96) was significantly higher than that of imaging diagnosis using Gd-EOB-MRI set (*A*_z_ = 0.86) or DWI set (*A*_z_ = 0.80) alone in differentiation of sHCC from DNs (*P* = 0.008 and 0.025, respectively). The combination of Gd-EOB-MRI and DWI showed a greater sensitivity (95.6%) but reduced specificity (66.7%). The specificity of TA (92.6%) was significantly higher than that of the combined set (*P* < 0.001), but no significant difference was observed in sensitivity (97.8 vs. 95.6%, *P* = 0.559).

**Conclusion:** TA-based T2WI showed a better classification performance than that of qualitative diagnosis using Gd-EOB-MRI and DW imaging in differentiation of sHCCs from DNs in cirrhotic liver. TA-based MRI may become a potential imaging biomarker for the early differentiation HCCs from DNs in cirrhosis.

## Introduction

Hepatocellular carcinoma (HCC) is one of the most common malignancies; almost 80% of HCC occurs in patients with cirrhosis (1, 2). Hepatocarcinogenesis in cirrhosis usually shows a multistep progression from benign nodules, early HCC, and progressive HCC (3). Early detection of HCC, differentiation from benign cirrhotic nodules, provides the greatest chance for long-term survival (4). However, a complete characterization of these nodules still remains a difficult diagnostic dilemma due to the overlap of imaging features (5, 6).

Based on the criteria of the American Association for the Study of Liver Diseases, arterial enhancement followed by later (portal or equilibrium phase) washout is defined as a conclusive diagnosis for HCC (7). However, this typical enhancement pattern is not always presented, especially for some well-differentiated or small HCCs (8, 9). Diffusion-weighted imaging (DWI) can provide additional value to routine dynamic MRI by improving the diagnostic sensitivity (10, 11). The restricted diffusion facilitates HCC diagnosis by reflecting tissue hypercellularity (12). Nevertheless, some small HCCs may not show restricted diffusion (13, 14).

Recently, as a hepatocyte-specific intake agent MR imaging, gadoxetic acid-enhanced MR imaging (Gd-EOB-MRI) provides both early dynamic vascular phase and delayed hepatobiliary phase (HBP) information, which has been increasingly applied in the characterization of liver nodules. Gd-EOB-MRI has been demonstrated a higher sensitivity for detecting HCCs than conventional dynamic MRI due to hypointensity on HBP images (5, 15, 16). However, some small HCCs may not show hypointensity on HBP images; in contrast, some DNs are hypointensity (5, 17, 18).

Texture analysis (TA) based on medical images is a postprocessing approach in differential diagnosis of benign and malignant diseases (19). TA based on MRI has been used for distinguishing breast cancer from normal tissue and classifying histological types (20), e.g., differentiating prostate cancer from normal tissue and classifying prostate cancers with different Gleason scores (21). In liver assessments, texture-based MRI can be used to differentiate different single liver lesions (22, 23), evaluate hepatic fibrosis and cirrhosis grades (24), and predict the HCC histological grade (25).

The value of TA-based MRI for discriminating cirrhotic nodules remains unclear; we hypothesized that MRI-based TA may be helpful to distinguish HCCs from DNs. Thus, we performed this study to estimate the feasibility of TA-based T2-weighted images in the differentiation of sHCC from DNs in cirrhotic liver.

## Materials and Methods

### Patient Samples

**Figure 1:**
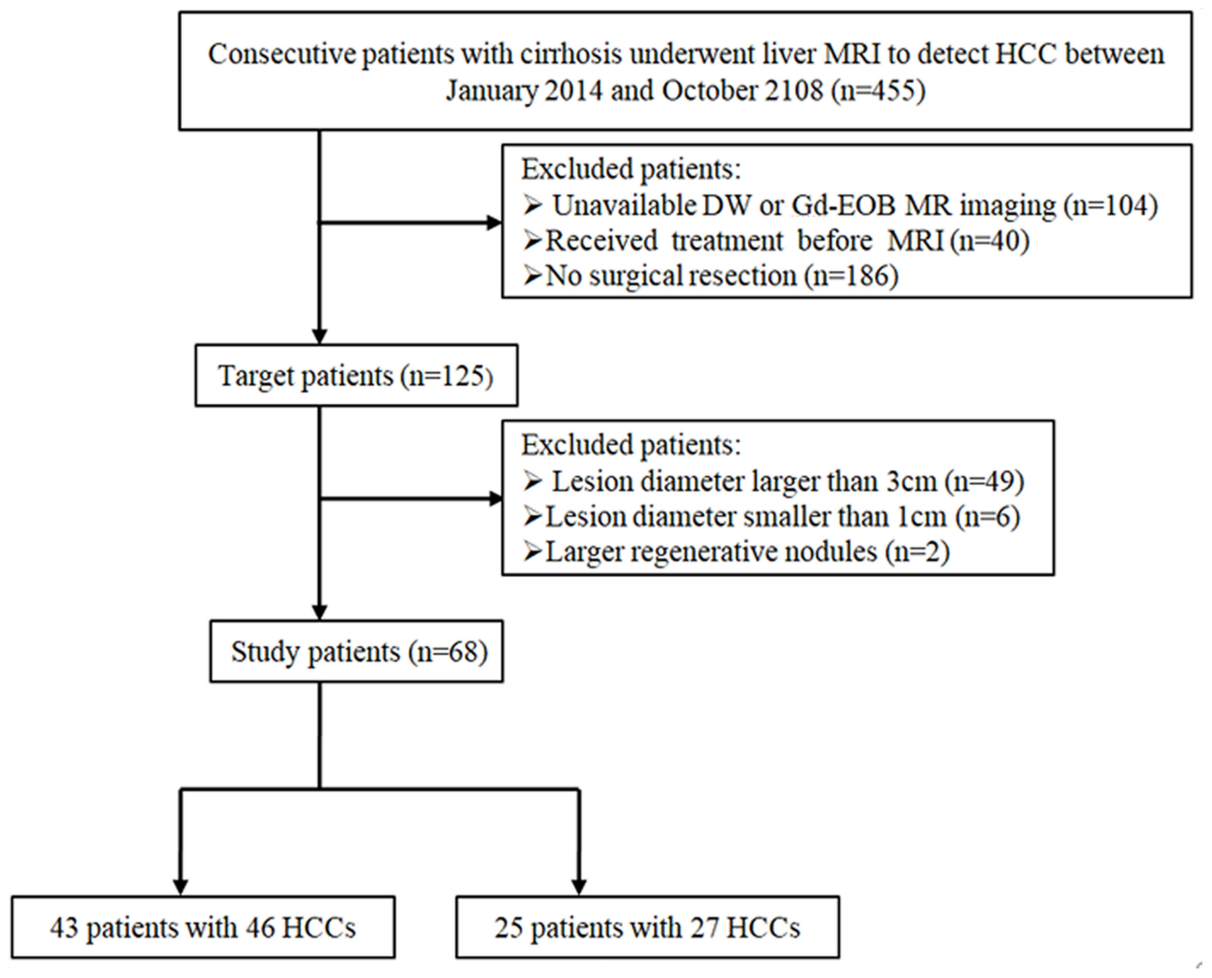
Flowchart of the study population.

This retrospective study was approved by the institutional review board of our hospital, and patient's informed consent was waived. We reviewed 455 consecutive patients with cirrhosis who underwent liver MRI to exclude HCC between January 2015 and October 2018. The inclusion criteria were as follows: (1) pathologically proven HCCs or DNs by surgical resection, (2) nodule diameter smaller than 3 cm and larger than 1 cm, (3) underwent both DW and Gd-EOB-MRI, and (4) received no treatment before MRI. Based on the inclusion criteria, ultimately a total of 68 patients [42 men, 26 women; median, 56 years (range, 30–73 years)] with 73 liver nodules (46 HCCs, 27 DNs) were enrolled. The patient inclusion flowchart is shown in Figure 1.

### MRI Acquisition

MR imaging was performed in a 3.0-T whole-body MR system (Achieva; Philips Healthcare, Best, Netherlands) with a 16-channel phased-array coil. The MRI protocol consisted of a respiratory-triggered T1-weighted turbo field-echo in-phase and opposed sequence [TR/first echo TE and second echo TE, 10/2.5 ms (in-phase) and 3.55 ms (opposed-phase); flip angle, 10°; matrix, 256 × 224; bandwidth, 434.3 Hz/pixel] and a breath-hold fat-saturated T2-weighted fast spin-echo sequence (TR/TE, 2,096/72 ms; flip angle, 90°; matrix size, 324 × 256; bandwidth, 258.4 Hz/pixel) with a 5-mm section thickness and a field of view (FOV) of 30–38 cm. DWI was performed using a respiratory triggering single-shot echo planar imaging sequence with *b* values of 0 and 800 s/mm^2^, spectral presaturation with inversion recovery for fat suppression, using a TR/TE of 1,600/70, matrix size of 100 × 100, acceleration factor of SENSE of 4.0, FOV of 30–35 cm, slice thickness of 5 mm, slice gap of 1 mm, and 33 axial slices. For Gd-EOB-MRI, unenhanced, arterial-phase (20–35 s), portal-phase (60 s), late-phase (3 min), and 20-min delayed HBP images were obtained using a T1-weighted three-dimensional (3D) turbo-field-echo sequence (T1 high-resolution isotropic volume examination; Philips Healthcare) (3.1/1.5; flip angle, 10°; matrix size, 228 × 211; bandwidth, 724.1 Hz/pixel) with a 2-mm section thickness and an FOV of 32–38 cm. The contrast agent was automatically administered intravenously at a rate of 1 ml/s for a dose of 0.025 mmol/kg body weight using a power injector, followed by a 20-ml saline flush.

### Image Analysis

All images were analyzed separately and independently reviewed by two radiologists (B.G.L and P.Y.Y, with 15 and 10 years' experience of liver MR imaging, respectively) who were blinded to the patients' clinical data and the pathological diagnosis. Three image sets were reviewed, respectively: a Gd-EOB-MRI set (precontrast T1- and T2-weighted images and arterial, portal, equilibrium, and HBP images), a DWI set (precontrast T1- and T2-weighted images and DW images), and combined sets. Four-week interval between image reviews was performed for the three reviewing sessions to avert any recall bias. The signal intensity (SI) of each lesion was evaluated on Gd-EOB-MR and DW images. The SI features were classified into three groups: hypointensity, isointensity, or hyperintensity compared to the surrounding liver parenchyma.

As described in previous studies (5, 15, 26), in Gd-EOB-MRI set, the diagnostic criteria for HCC were defined as follows: (a) a nodule showed typical enhancement pattern (arterial enhancement and late portal or equilibrium washout); (b) a nodule with arterial enhancement without later washout, but hypointensity on HBP images, or peripheral rim enhancement on the late dynamic phase images (capsular appearance); and (c) a nodule without arterial enhancement, but larger than 1.5 cm and showed hypointensity on HBP images. In the DW set, if a lesion showed hyperintensity on DW images, it was interpreted as an HCC (14). In combined sets, if a lesion satisfied the HCC criteria of Gd-EOB-MRI or DWI, it would be identified as an HCC.

### Texture Analysis

#### Texture Calculation

**Figure 2:**
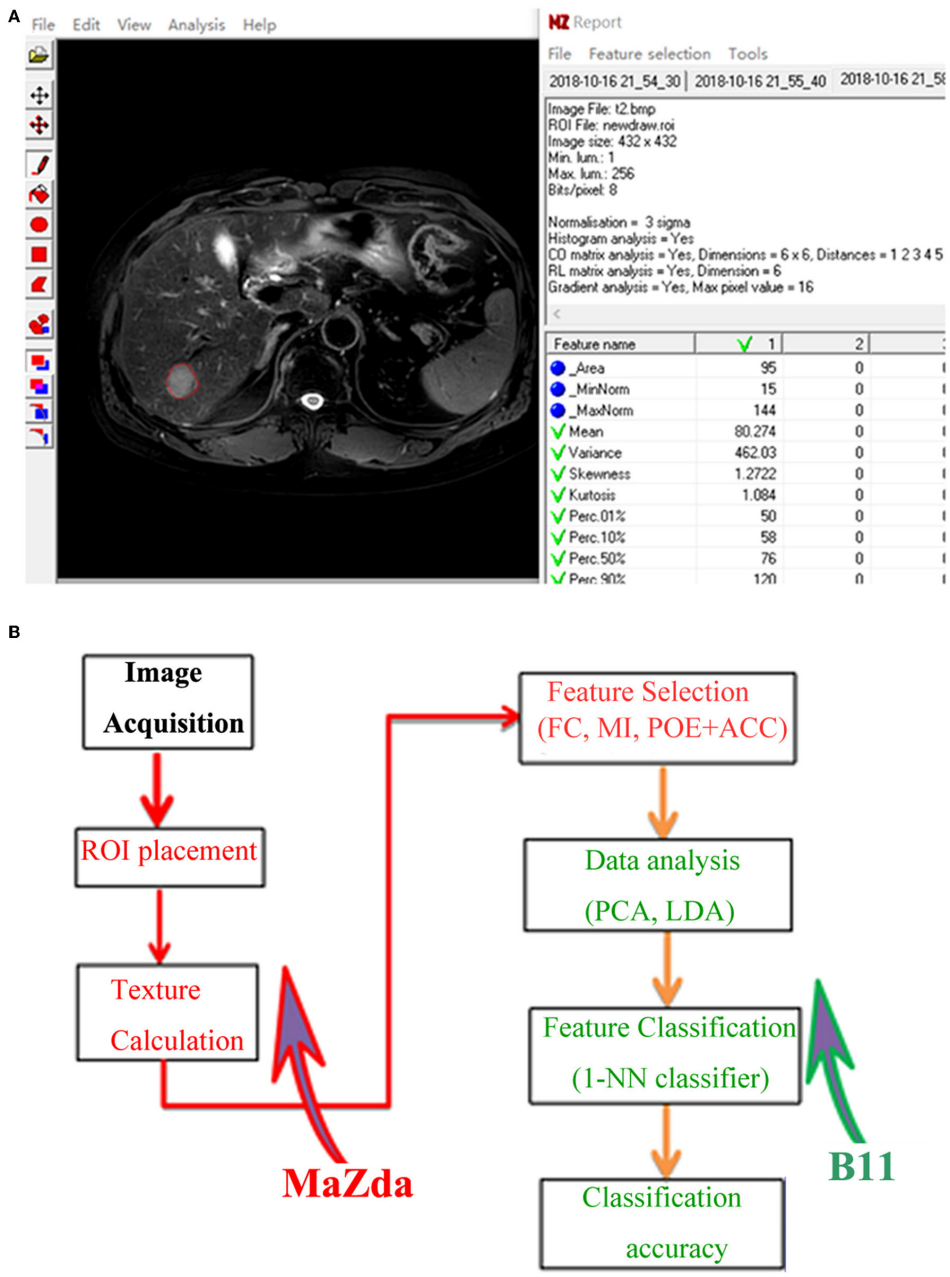
**(A)** Texture calculation for a 63-year-old man with a pathologically proven hepatocellular carcinoma (HCC) on fat-saturated T2-weighted images by using MaZda. A single region of interest (ROI) was defined and delineated on the image section depicting the maximum lesion diameter. **(B)** Procedure of texture analysis: texture calculation and feature selection were performed using MaZda; data analysis and classification were performed using program B11. ROI, region of interest; FC, Fisher coefficients; MI, mutual information; POE + ACC, minimization of both classification error probability and average correlation coefficients; PCA, principal component analysis; LDA, linear discriminant analysis; 1-NN, *first* nearest neighbor.

The axial FS T2-weighted images were exported in “.dicom” format from the PACS for texture analysis. One of the radiologists (X.Z) manually segmented images for each lesion using a free open-source software package MaZda 4.6 (URL: http://www.eletel.p.lodz.pl/programy/mazda/), and a single region of interest (ROI) was defined and delineated on the image section depicting the maximum lesion diameter (Figure 2A). Seven lesions (two HCCs and five DNs) were isointense on FS-T2-WI, in this case, T1-weighted or gadoxetic acid-enhanced images were used for accurate ROI placement. Refer to previous studies (20, 23), ROI gray-level normalization was performed by adjusting image intensities in the range of *u* ± 3σ (where *u* is the gray-level mean and σ is the gray-level standard deviation). A total of 279 texture parameters that derived from six statistical image descriptors were computed for each ROI (Table 1).

**Table 1:** Texture parameters calculated in MaZda.

**Computational methods**	**Number**	**Texture parameters**
Histogram	9	Mean, variance, skewness, kurtosis, percentiles 1, 10, 50, 90, and 99%
Co-occurrence matrix	220	Angular second moment, contrast, correlation, sum of squares, inverse difference moment, sum average, sum variance, sum entropy, entropy, difference variance, and difference entropy. Features are computed for 5 between-pixels distances (1, 2, 3, 4, and 5) and for four various directions (vertical, horizontal, 0, and 135).
Run-length matrix	20	Run-length non-uniformity, gray-level non-uniformity, long-run emphasis, short run emphasis, and fraction of image in runs. Features are computed for four various directions (vertical, horizontal, 0, and 135).
Absolute gradient	5	Mean, variance, skewness, kurtosis, and percentage of pixels with nonzero gradient
Autoregressive model	5	Teta1 (θ1), Teta2 (θ2), Teta3 (θ3), Teta4 (θ4), and Sigma (σ)
Wavelet (*n* = 20)	20	WavEn (wavelet energy). Feature is computed at five scales within four frequency bands LL, LH, HL, and HH.

#### Feature Selection

To determine the most discriminative texture features for differentiating sHCCs from DNs, as mentioned previously (23), we used three texture feature selection methods, including Fisher coefficients, minimization of both classification error probability and average correlation coefficients (POE + ACC), and mutual information (MI), respectively.

#### Feature Classification

Feature classification was performed in a statistical program B11 (version 4.6). As described in a previous study (27), principal component analysis (PCA) and linear discriminant analysis (LDA) were used for reducing the feature vector dimension and increasing the discriminative power. Then, the first nearest neighbor (1-NN) classifier with feature vector standardization was applied to determine classification accuracy (23, 24). The procedure of TA is shown in Figure 2B.

### Histopathology Evaluation

International Working Party criteria was used for the evaluation of hepatocellular nodular (28). DNs were defined as a lesion with hepatocytes dysplasia but no definite histological features of malignancy, which were classified as low- or high-grade based on the cytological and architectural atypia (29).

### Statistical Analysis

The sensitivity and specificity for differentiation of sHCCs from DNs were calculated for qualitative diagnosis and TA. The overall diagnostic efficiency was evaluated by calculating area under the receiver operating characteristic (ROC) curve (*A*_z_), and the ROC curves were plotted based on the dichotomous classification results of each diagnostic approach, and the diagonal segments are produced by ties. Mann–Whitney and chi-square test (or Fisher test) were performed for quantitative and categorical variables, respectively. All the statistical tests were performed using SPSS 16.0 (SPSS Inc., Chicago, IL, USA) package, and statistical significance was accepted for *P* < 0.05.

## Result

### Patient Characteristics

Patients' characteristics are summarized in Table 2. Of the 68 patients, 63.2% (43/68) with 46 lesions were diagnosed with HCCs (diameter range, 1.2–3.0 cm; mean, 1.9 cm), and 36.8% (25/68) of patients with 27 nodules were diagnosed with DNs (diameter range, 1.0–2.9 cm; mean, 1.7 cm). Of the 27 DNs, 13 were high-grade DNs (HGDNs) and 14 were low-grade DNs (LGDNs).

**Table 2:** Patients' characteristics.

**Parameters**	**Value**
Patient number	68
Age median [range] (years)	56 (30–73)
Male/female	42/26
Child-pugh	
A	50
B	12
C	6
AFP serum >200 ng/ml	11
AFP serum <200 ng/ml	51
AFP unobtainable	6
Etiology of liver cirrhosis^*^	
HBV	51
HCV	13
Ethanol	8
Others	2

AFP, Alpha-fetoprotein;

* *A patient could have multiple etiologies*.

### Diagnostic Performance of Qualitative MRI Diagnosis

The SI features of HCCs and DNs on Gd-EOB-MRI and DWI are shown in Table 3. The diagnostic performance of each imaging set for differentiating sHCCs from DNs are shown in Table 4.

**Table 3:** Signal features of hepatocellular nodules.

**SI features**	**HCCs (*n* = 46)**	**DNs (*n* = 27)**	**HGDN (*n* = 13)**
**DWI**			
Hypointensity	0 (0%)	4 (14.8%)	2 (15.4%)
Isointensity	5 (10.9%)	15 (55.6%)	4 (30.8%)
Hyperintensity	41 (89.1%)	8 (29.6%)	7 (53.8%)
**Arterial phase**			
Hypointensity	6 (13.1%)	4 (14.8%)	1 (7.7%)
Isointensity	10 (21.7%)	10 (37.0%)	4 (30.8%)
Hyperintensity	30 (65.2%)	13 (48.1%)	8 (61.5%)
**Portal phase**			
Hypointensity	30 (65.2%)	4 (14.8%)	1 (7.7%)
Isointensity	4 (8.7%)	8 (29.6%)	2 (15.4%)
Hyperintensity	12 (26.1%)	15 (55.6%)	10 (76.9%)
**Equilibrium phase**			
Hypointensity	36 (78.3%)	6 (22.2%)	3 (23.0%)
Isointensity	6 (13.0%)	16 (59.2%)	5 (38.5%)
Hyperintensity	4 (8.7%)	5 (18.6%)	5 (38.5%)
**HBPI**			
Hypointensity	40 (87.0%)	3 (11.1%)	2 (15.4%)
Isointensity	5 (10.9%)	19 (70.3%)	7 (53.8%)
Hyperintensity	1 (2.1%)	5 (18.6%)	4 (30.8%)

*SI, signal intensity; HCC, Hepatocellular carcinoma; DNs, Dysplastic nodules; HGDN, high-grade dysplastic nodules; DW, Diffusion-weighted imaging; HBPI, Hepatobiliary phase imaging*.

**Table 4:** Diagnostic performance of DW and gadoxetic acid-enhanced imaging.

**Imaging sets**	**A_**z**_ [95% CI]**	**Sensitivity**	**Specificity**	**Accuracy**
Gd-EOB-MRI set	0.86 [0.76, 0.95]	82.6% (38/46)	88.9% (24/27)	84.9% (62/73)
DWI set	0.80 [0.68, 0.91]	89.1% (41/46)	70.3% (19/27)	82.2% (60/73)
Combined sets	0.81 [0.69, 0.93]	95.6% (44/46)	66.7% (18/27)	84.9% (62/73)

**Figure 3:**
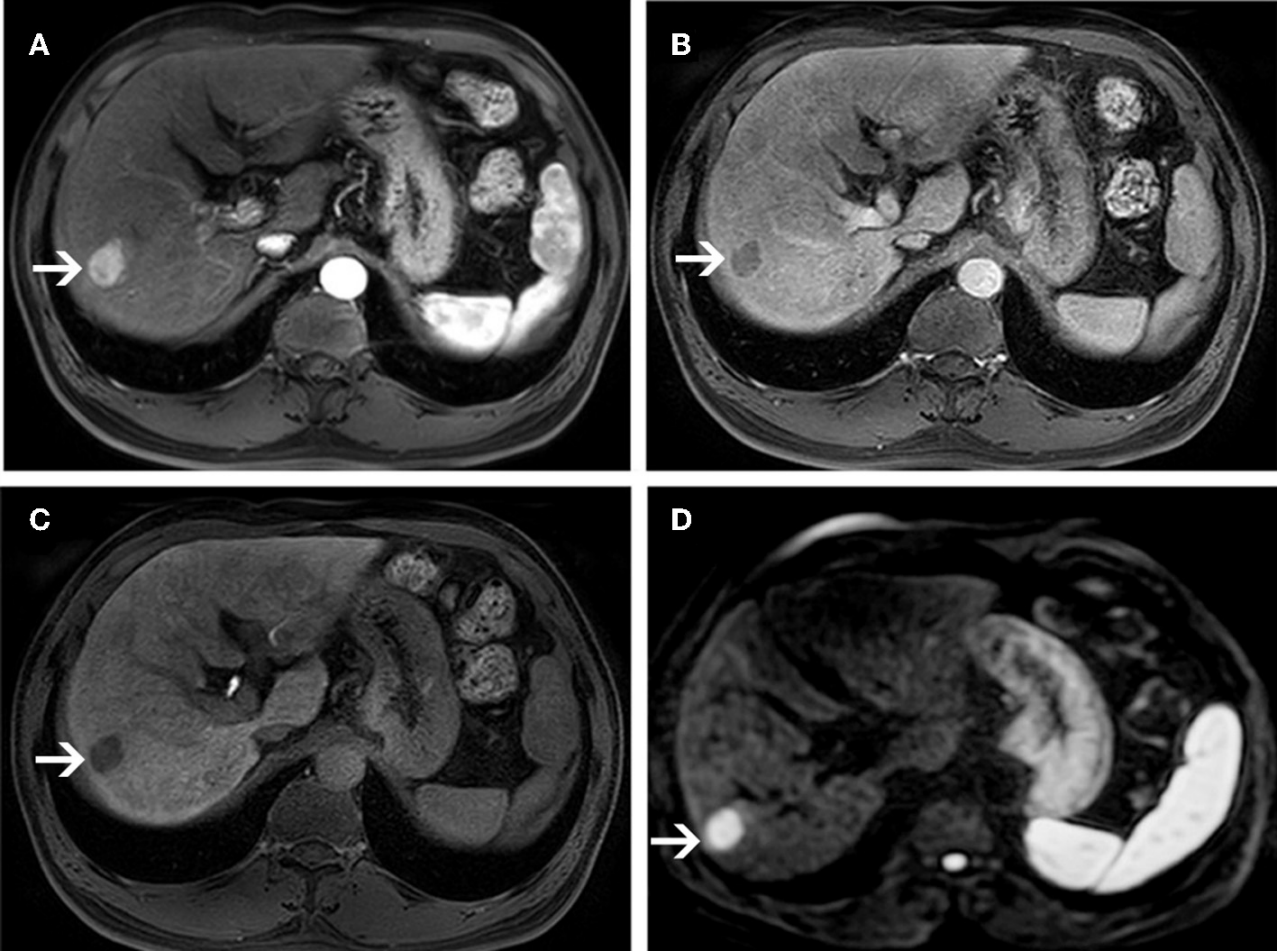
MR images of a 58-year-old man with a pathologically proven HCC (white arrows) and a history of hepatitis B virus infection. An arterial-phase image **(A)** shows an enhancing nodule in segment VI of the liver. An equilibrium phase MR image **(B)** shows a nodule demonstrating washout of contrast material, and showing capsular appearance. At the hepatobiliary phase **(C)**, the lesion is hypointensity compared to the surrounding liver parenchyma. On diffusion-weighted image **(D)**, the lesion is hyperintensity compared to the surrounding liver parenchyma.

**Figure 4:**
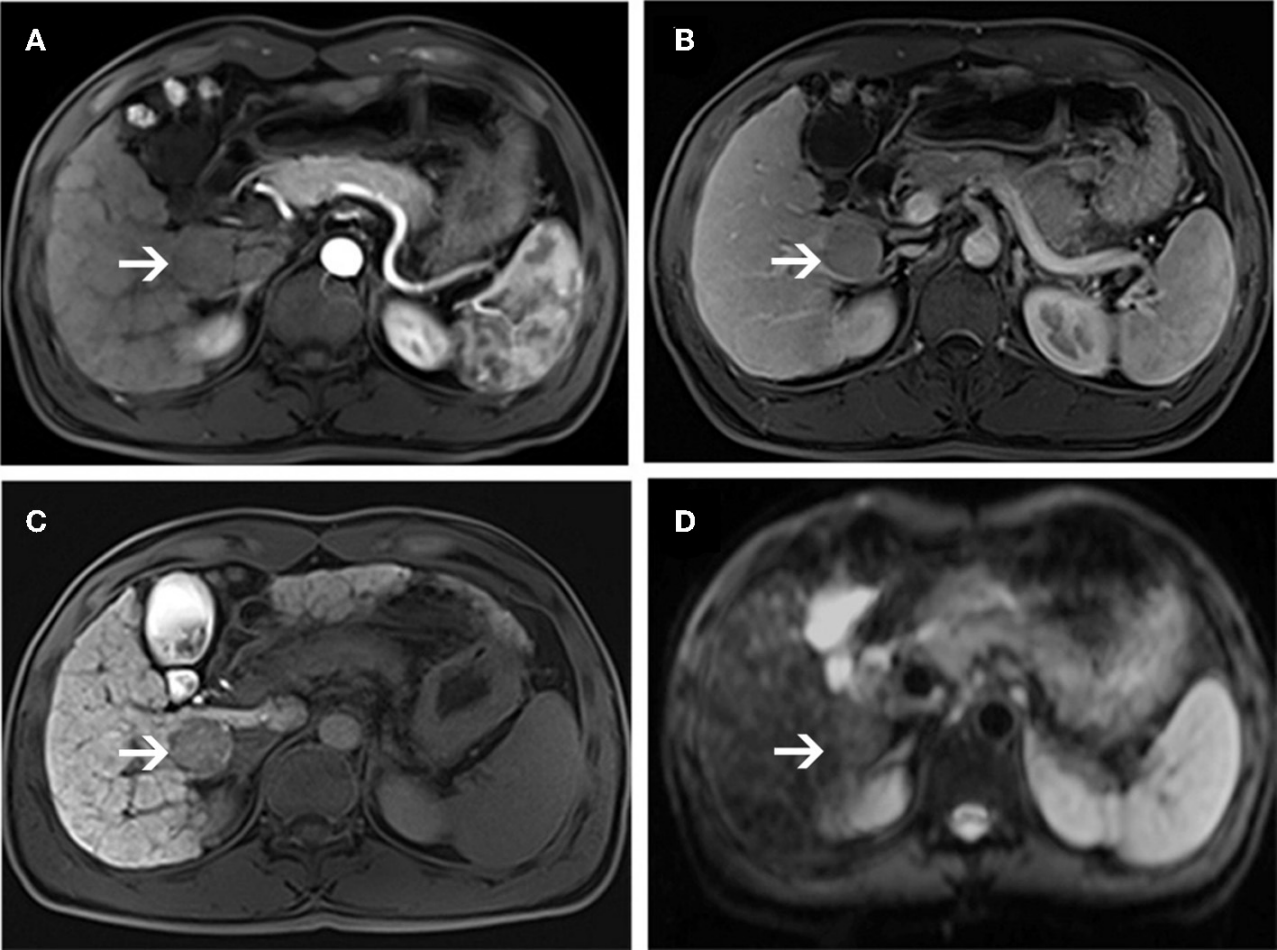
MR images of a 66-year-old woman with a pathologically proven HCC (white arrows) and a history of hepatitis C virus infection. An arterial-phase image **(A)** shows a hypovascular nodule in segment I of the liver. An equilibrium phase MR image **(B)** shows a slightly hypointensity nodule compared to the surrounding liver parenchyma. A hepatobiliary phase image **(C)** shows a hypointensity lesion compared to the surrounding liver parenchyma. On diffusion-weighted image **(D)**, the lesion is nearly isointensity compared to the surrounding liver parenchyma.

**Figure 5:**
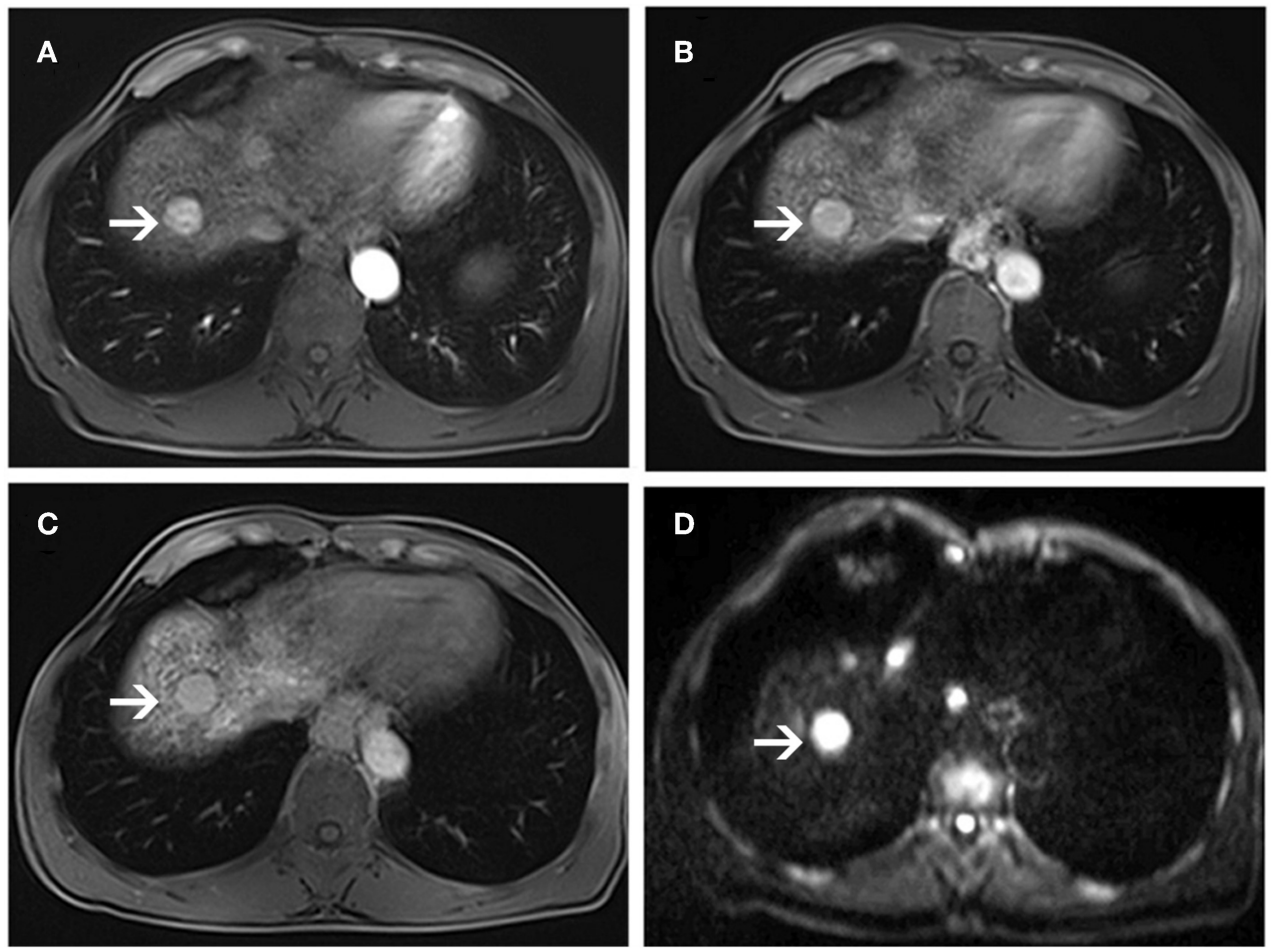
MR images of a 56-year-old man with a pathologically proven high-grade dysplastic nodule (white arrows) and a history of hepatitis B virus infection. Arterial-phase image **(A)** shows an enhancing nodule in segment VIII of the liver. Equilibrium-phase MR image **(B)** shows a nodule not demonstrating washout of the contrast material. A hepatobiliary phase image **(C)** shows nearly isointensity compared to the surrounding liver parenchyma. On diffusion-weighted image **(D)**, the lesion shows hyperintensity compared to the surrounding liver parenchyma.

In Gd-EOB-MRI set, among the 46 sHCCs, 50% (23/46) of lesions showed typical enhancement patterns (Figure 3): 13 lesions showed arterial enhancement without late washout but hypointensity on HBP images (Supplementary Figure 1), 2 nodules showed hypovascular on dynamic study, but larger than 1.5 cm and showed hypointensity on HBP images (Figure 4), and the other 8 nodules satisfied none of the Gd-EOB-MRI criteria for HCC (Supplementary Figure 2). Of the 27 DNs, no nodule showed atypical enhancement patterns, 24 nodules (11 HGDNs, 13 LGDNs) showed iso/hyperintensity on HBP images (Figure 5), and 3 lesions (2 HGDNs, 1 LGDN) showed arterial enhancement and hypointensity on HBP images. The sensitivity and specificity for differentiating sHCCs from DNs in Gd-EOB-MRI set were 82.6% (38/46) and 88.9% (24/27), respectively.

In DWI set, 41 nodules among the 46 sHCCs showed hyperintensity on DWI (Figure 3), and 5 nodules showed iso/hypointensity on DWI (Figure 4). Of the 27 DNs, 19 nodules (6 HGDNs, 13 LGDNs) showed iso/hypointensity on DWI (Supplementary Figure 3), and 8 nodules (6 HGDNs, 2 LGDNs) showed hyperintensity on DWI (Figure 5). The sensitivity and specificity for identifying sHCCs from DNs in DWI set were 89.1% (41/46) and 70.3% (19/27), respectively.

In combined sets, of the 46 sHCCs, only 2 nodules were mistaken for DNs. Among the 27 DNs, 18 nodules (7 HGDNs, 11 LGDNs) were diagnosed accurately. Consequently, the sensitivity and specificity for differentiating sHCCs from DNs in combined sets was 95.6% (44/46) and 66.7% (18/27), respectively.

### TA Results

Texture subsets based on MI and Fisher coefficients were frequently derived from the co-occurrence matrix, whereas texture features created using the POE + ACC method were frequently derived from wavelet (Table 5).

**Table 5:** Texture feature subsets best-suited for the discrimination of w-HCCs and DNs on T2-W images, according to Fisher coefficient, the PEO+ACC, and Mutual information.

**Feature rank**	**Fisher coefficient**	**POE + ACC**	**Mutual information**
1	WavEnLL_s-1	WavEnHH_s-3	WavEnLL_s-2
2	WavEnLL_s-2	WavEnLH_s-3	WavEnLL_s-1
3	S(0,1)SumOfSqs	WavEnLL_s-3	S(0,5)SumAverg
4	S(0,1)SumAverg	WavEnHH_s-2	S(0,3)SumOfSqs
5	S(0,1)SumVarnc	WavEnLH_s-2	S(0,2)SumOfSqs
6	S(1,0)SumOfSqs	WavEnLL_s-2	S(1,-1)SumVarnc
7	S(1,0)SumVarnc	WavEnLH_s-1	S(1,-1)SumAverg
8	S(0,2)SumAverg	WavEnLL_s-1	S(0,1)SumAverg
9	S(1,-1)SumAverg	Vertl_LngREmph	S(0,1)SumOfSqs
10	S(0,3)SumAverg	S(0,1)SumAverg	S(1,0)SumOfSqs

*POE + ACC, minimization of both classification error probability and average correlation coefficients; WavEn, Wavelet energy; SumOfSqs, Sum of squares; SumAverg, Sum average; SumVarnc, Sum variance; Vertl_LngREmph, Vertical long-run emphasis*.

Fisher coefficients, POE + ACC, and MI methods resulted in a similar misclassification rate of 4.1–6.8, 4.1–6.8, and 4.1–5.5%, respectively (Table 6). In terms of feature classification, PCA and LDA resulted in an equivalent misclassification rate of 4.1–6.8 and 4.1–5.5%, respectively (Table 6).

**Table 6:** Diagnostic performance of texture analysis.

**Feature selection method**	**TA Method**	**Misclassified**	**A_**z**_**	**Sensitivity**	**Specificity**	**Accuracy**
		**rates**	**[95% CI]**			
Fisher coefficient	PCA	5/73 (6.8%)	0.94 [0.87, 1]	97.8% (45/46)	85.2% (23/27)	93.2% (68/73)
	LDA	3/73 (4.1%)	0.96 [0.91, 1]	97.8% (45/46)	92.6% (25/27)	95.9% (70/73)
POE + ACC	PCA	4/73 (5.5%)	0.94 [0.87, 1]	95.7% (44/46)	92.6% (25/27)	94.5% (69/73)
MI	LDA	5/73 (6.8%)	0.93 [0.85, 1]	95.7% (44/46)	88.9% (24/27)	93.2% (68/73)
	PCA	3/73 (4.1%)	0.96 [0.91, 1]	97.8% (45/46)	92.6% (25/27)	95.9% (70/73)
	LDA	4/73 (5.5%)	0.94 [0.87, 1]	95.7% (44/46)	92.6% (25/27)	94.5% (69/73)

*POE + ACC, Minimization of both classification error probability and average correlation coefficients; MI, Mutual information; PCA, Principle component analysis; LDA, Linear discriminant analysis*.

**Figure 6:**
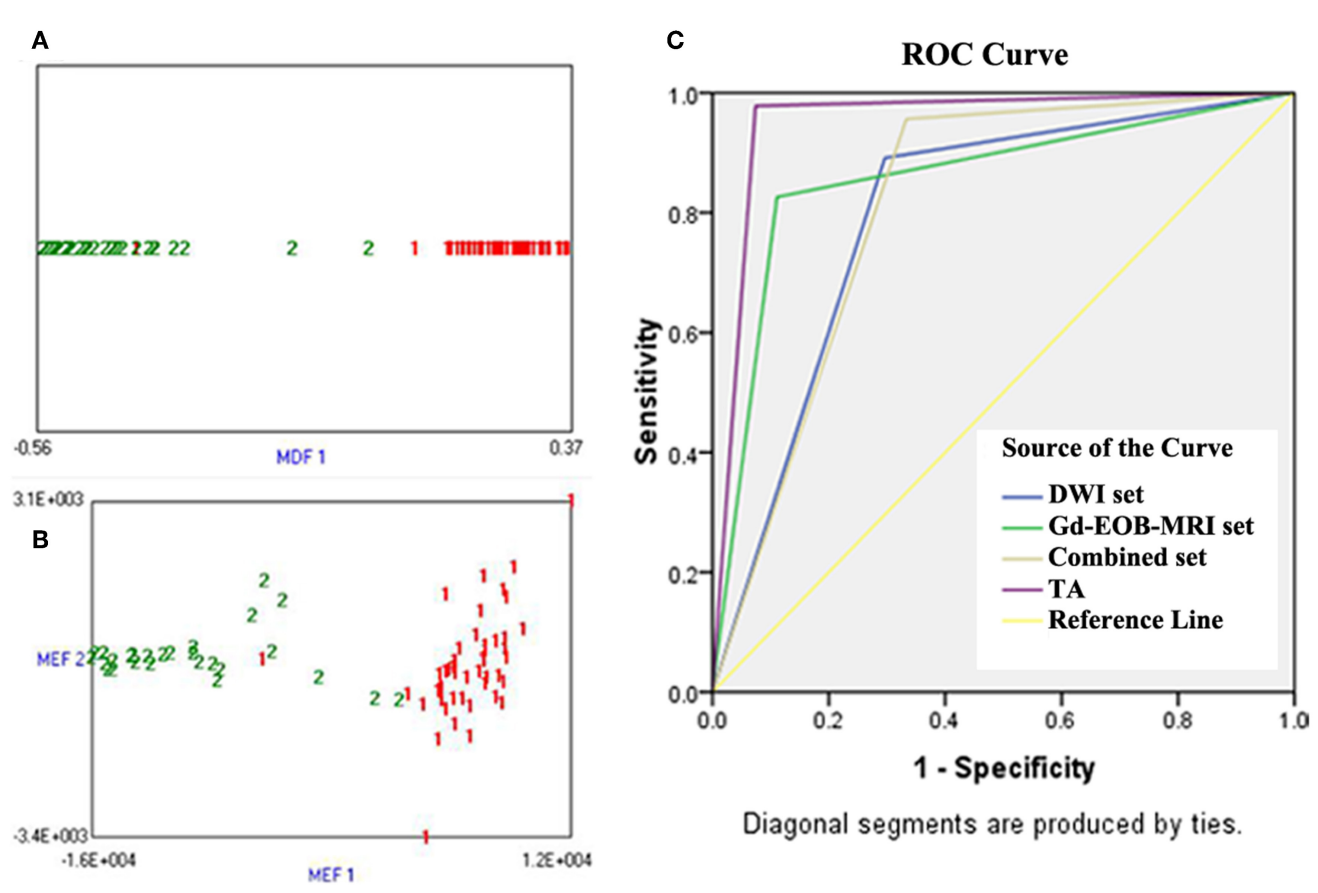
**(A)** Linear discriminant analysis (LDA) combining Fisher coefficient for classifying small hepatocellular carcinoma (sHCC) and dysplastic nodules (DNs), 1 (red) represents sHCC and 2 (green) represents DNs; it shows that one sHCC was misclassified as a DN, and two DNs were misclassified as sHCC. **(B)** Principal component analysis (PCA) combining MI for classifying sHCC and DNs, 1 (red) represents sHCC and 2 (green) represents DNs; it shows that one sHCC was misclassified as a DN, and two DNs were misclassified as sHCC. **(C)** Receiver operating characteristic (ROC) curves of imaging sets and TA for the differentiation of sHCC from DNs, the ROC curves were plotted based on the dichotomous classification results of each diagnostic approach, and the diagonal segments are produced by ties. The position of the “kink” of curves represented diagnostic efficacy; *Y*-axis represented sensitivity, and *X*-axis represented 1-specificity.

Both LDA combining Fisher coefficients and PCA combining MI Fisher showed the lowest misclassification rate of 4.1% (3/73). Only one HCC was misclassified as a DN, and two DNs were misclassified as HCCs (Figures 6A,B). With regard to the ROC analysis, TA demonstrated an *A*_z_, sensitivity, specificity, and accuracy of 0.96 (95% CI: 0.91, 1), 97.8% (45/46), 92.6% (25/27), and 95.9% (70/73), respectively (Table 5).

### Comparison of Diagnostic Performance

The ROC curve of each diagnostic method is shown in Figure 6C. The diagnostic performance of TA (*A*_z_ = 0.96, 95% CI: 0.91, 1) was significantly higher than that of imaging diagnosis with DWI (*A*_z_ = 0.80, 95% CI: 0.68, 0.91) or Gd-EOB-MRI (*A*_z_ = 0.86, 95% CI: 0.76, 0.95) alone (*P* = 0.008 and 0.025, respectively). The specificity of TA (92.6%) was significantly higher than that of DWI and Gd-EOB-MRI combined (66.7%) (*P* < 0.001), but no significant difference was observed in sensitivity (97.8 vs. 95.6%; *P* = 0.559).

## Discussion

This study aimed to identify whether MRI-based TA can be used to distinguish sHCC from DNs in cirrhotic liver. We also compared the performance of TA with DWI and Gd-EOB-MRI. The findings showed that TA-based T2WI had a satisfactory diagnostic value. The diagnostic efficacy of TA was significantly higher than that of qualitative diagnosis with DWI or Gd-EOB-MRI alone. Although the combination of DWI and Gd-EOB-MRI showed sensitivity equivalent to that of TA, TA showed significantly higher specificity than that of the combination qualitative diagnosis.

In the present study, we found that only 50% of sHCCs fit the American Association for the Study of Liver Diseases criteria, but HBP imaging improved the detection of sHCC; up to 15 sHCCs with atypical enhancement were detected by hypointensity on HBP images. In Gd-EOB-MRI set, the sensitivity and specificity were 82.6 and 88.9%, respectively, which is similar to the 85% sensitivity but significantly higher than the 42% specificity on imaging using gadoxetic acid disodium (14), and is similar to the 92% specificity but higher than the 71% sensitivity on imaging using gadobenate dimeglumine (30). Furthermore, Gd-EOB-MRI yields a better specificity than that of DWI set, with a specificity 18.6% greater than that of DWI, which is inconsistent with one study (14) in which Gd-EOB-MRI showed lower specificity than DWI for differentiating HCC from benign hepatic nodules. Nevertheless, we still found that 13.4% of sHCCs did not show hypointensity on 20-mine HBP images, which may be related to the overexpression of organic anionic transporting polypeptide 8 (OATP-8) in tumors; about 5–12% of small HCCs overexpress organic anionic transporting polypeptide 8 (18).

In DWI set, we found that the overall sensitivity in the identification of sHCCs and DNs was almost 89.1%, which was supported by previous reports showing that 81–88% of sHCCs showed hyperintensity on DW images (14, 31). Nevertheless, our study showed a relatively low specificity of 70.3% compared with some previous studies that reported specificity values of 79.0–94.4% (32, 33), which may be attributed to six HGDNs that showed some imaging features supporting HCC, such as hyperintensity on arterial phase without washout, hypointensity on HBP images, and/or hyperintensity on DWI.

In our study, the combination DW and Gd-EOB-MRI demonstrated an increase in the sensitivity for diagnosing sHCC compared with each imaging modality alone. Thus, the results are concordant with the previous data reported by Park et al. (34). However, our study resulted in a lower specificity of only 66.7%. Other than the expected variation between observers and institutions, the difference might be attributable to the fact that the benign hepatic nodules included in our study were all DNs.

The feasibility of TA in the classification of liver lesions has been widely discussed in CT and MRI (22, 23, 25, 35, 36). It is well-known that ROI placement plays a key role for TA; if a lesion shows isointensity, it may be difficult to place an ROI accurately. In this study, relatively large number of liver nodules showed isointensity on both DWI and dynamic Gd-EOB-MRI. Thus, this study analyzed the value of TA based on T2W-MRI images in discrimination of sHCC from DNs in cirrhosis. To our knowledge, this is the first study to assess whether HCCs and DNs in cirrhosis can be fully classified using TA. In comparison with previous studies that used T2WI-based TA for classification of liver lesions, the misclassification rate by TA was 4.1%, which was lower than the 9.7% misclassification rate for distinguishing HCC from hepatic hemangioma and metastases (22) and the 12% misclassification rate for distinguishing liver cysts and hemangiomas (23). Furthermore, the primary advantage of our study was that we compared the diagnostic efficacy of TA with imaging diagnosis and found that TA showed better performance than that shown by imaging diagnosis with DW and gadoxetic acid-enhanced imaging alone. Although combined imaging strategy showed similar sensitivity as TA (95.6 vs. 97.8%) for identification of sHCC and DNs, the specificity of TA (92.6%) was significantly higher than that of the combined approach (63.0%).

In terms of misclassification rates, the performances of these feature selection methods (Fisher, POE + ACC, or MI) showed no clear superiority, supporting the results of previous studies (20, 23). For the MI and Fisher methods, the texture parameters resulting from the co-occurrence matrix were more frequently assigned to the feature subsets than parameters of any other category, thus supporting findings of previous studies. Interestingly, for POE + ACC selection, texture parameters resulting from the wavelet were more frequently assigned to the feature subsets than parameters of any other category.

This study had some limitations. First, we could not divide the study population into training and test datasets due to the relatively small size and because we were primarily interested in the feasibility of texture-based classification for identification of sHCCs and DNs in cirrhosis. Second, we did not assess the lesions that were not detected on MRI because the TA and imaging diagnosis is quite difficult to perform in those lesions, so it might cause the possibility of a bias at inclusion. Third, the performance of TA combined with qualitative diagnosis was not assessed because TA has showed significantly higher performance than qualitative imaging diagnosis. Actually, the combination of TA and qualitative diagnosis may improve performance, and it needs further researches to confirm the additional value of combination diagnosis.

In conclusion, this preliminary study demonstrates that MRI-based TA shows better classification performance than imaging diagnosis for discriminating sHCC from DNs in cirrhotic liver. Although promising, these results are preliminary and require verification using a larger and independent dataset to appraise their potential for clinical translation. After validation, texture-based MRI may become a potential imaging biomarker for early differentiating HCCs from DNs in cirrhosis.

## Data Availability Statement

All datasets generated for this study are included in the article/Supplementary Material.

## Ethics Statement

This retrospective study was approved by the institutional review board at Affiliated Cancer Hospital & Institute of Guangzhou Medical University, and the requirement of patients' informed consent was waived.

## Author Contributions

XZ and JL: conception and design. XZ and HT: manuscript writing. JY, JP, and PY: provision of study materials or patients. JY and JP: collection and assembly of data. BL and JL: MRI analysis and interpretation. XZ and HT: statistical analysis. XZ and JL: final approval of manuscript.

### Conflict of Interest

The authors declare that the research was conducted in the absence of any commercial or financial relationships that could be construed as a potential conflict of interest.
