# Supplementary material for: Differentiation of Small Hepatocellular Carcinoma From Dysplastic Nodules in Cirrhotic Liver: Texture Analysis Based on MRI Improved Performance in Comparison Over Gadoxetic Acid-Enhanced MR and Diffusion-Weighted Imaging
Source: Front Oncol. 2020 Jan 10;9:1382. doi: 10.3389/fonc.2019.01382 (PMC6966306; doi:10.3389/fonc.2019.01382)
Supplement: Supplementary file 1 [file Data_Sheet_1.pdf]

Supplementary Material

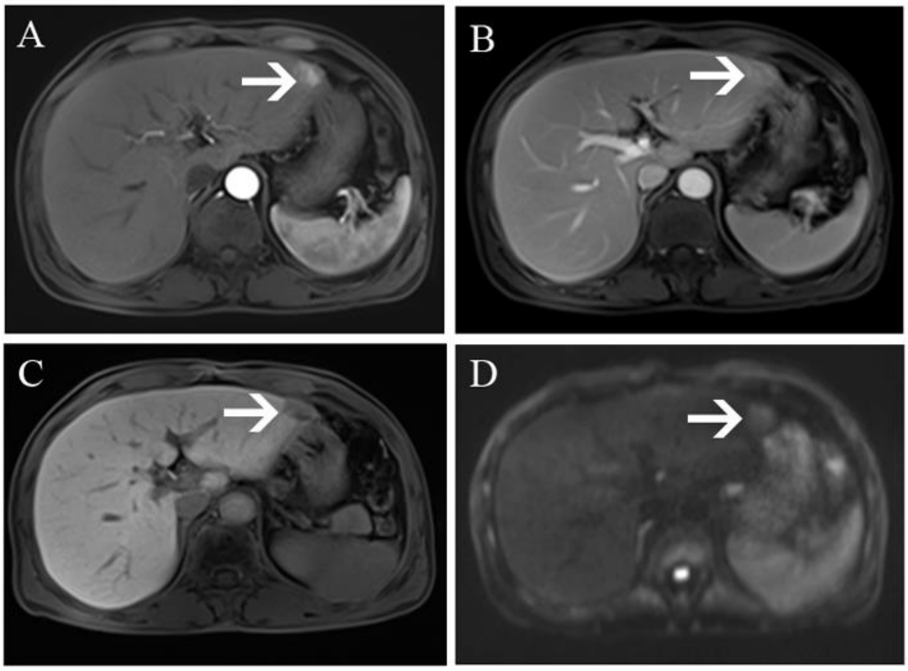

Supplementary FIGURE 1. MR images of a 63-year-old man with a pathologically proven HCC (white arrows) and a history of hepatitis C virus infection. Arterial-phase imaging shows an enhancing nodule in segment II of the liver (A). Equilibrium-phase MR image (B) shows a nodule not demonstrating washout of the contrast material. The lesion is hypointensity compared to the surrounding liver parenchyma (C). On diffusion-weighted image (D), the lesion shows hyperintensity compared to the surrounding liver parenchyma.

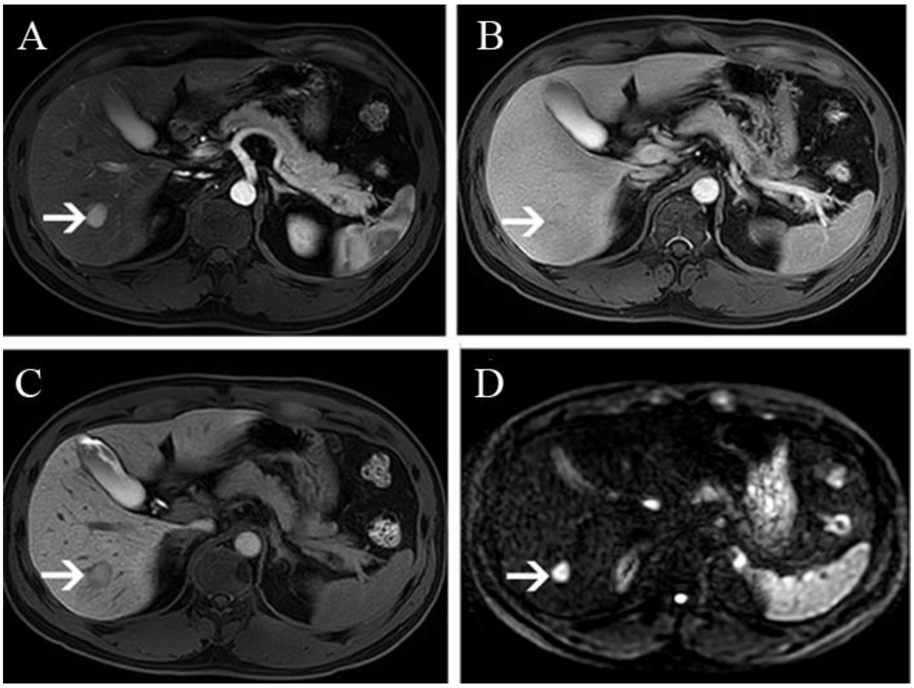

Supplementary FIGURE 2. MR images of a 43-year-old man with a pathologically proven HCC (white arrows) and a history of hepatitis B virus infection. Arterial-phase image (A) shows an enhancing nodule in segment VI of the liver. Equilibrium-phase MR image (B) shows a nodule not demonstrating washout of the contrast material. A hepatobiliary phase image (C) shows isointensity compared to the surrounding liver parenchyma. On diffusion-weighted image (D), the lesion shows hyperintensity compared to the surrounding liver parenchyma.

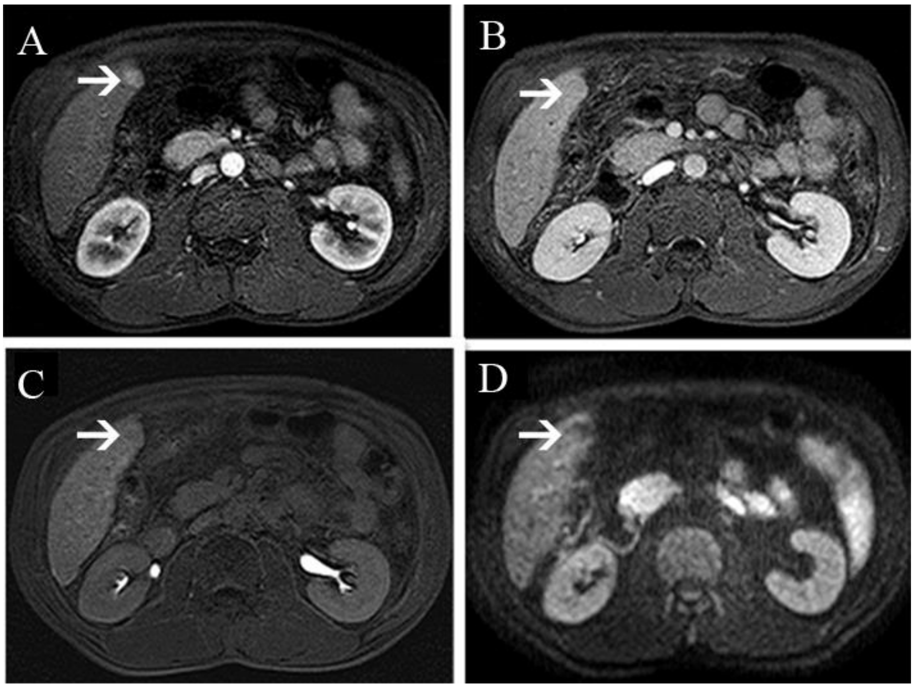

Supplementary FIGURE 3. MR images of a 47-year-old man with a pathologically proven Low-grade dysplastic nodule (white arrows) and a history of hepatitis B virus infection. Arterial-phase image (A) shows an enhancing nodule in segment V of the liver. Equilibrium-phase MR image (B) shows a nodule not demonstrating washout of the contrast material. A hepatobiliary phase image (C) shows a lesion nearly isointensity compared to the surrounding liver parenchyma. On diffusion-weighted image (D), the lesion shows iso to hypointensity compared with the surrounding liver parenchyma.
